# Hydrogen Desorption Properties of LiBH_4_/*x*LiAlH_4_ (*x* = 0.5, 1, 2) Composites

**DOI:** 10.3390/molecules24101861

**Published:** 2019-05-15

**Authors:** Qing He, Dongdong Zhu, Xiaocheng Wu, Duo Dong, Meng Xu, Zhaofei Tong

**Affiliations:** 1 Key Laboratory of Air-driven Equipment Technology of Zhejiang Province, Quzhou University, Quzhou 324000, China; helinqi@163.com (Q.H.); dongduohit@163.com (D.D.); xmm2021@163.com (M.X.); tzhaof@163.com (Z.T.); 2 Department of Materials Science and Engineering, Zhejiang University, Hangzhou 310058, China; 11026032@zju.edu.cn

**Keywords:** LiBH_4_LiAlH_4_, activation energy, hydrogen storage materials, dehydrogenation

## Abstract

A detailed analysis of the dehydrogenation mechanism of LiBH_4_/*x*LiAlH_4_ (*x* = 0.5, 1, 2) composites was performed by thermogravimetry (TG), differential scanning calorimetry (DSC), mass spectral analysis (MS), powder X-ray diffraction (XRD) and scanning electronic microscopy (SEM), along with kinetic investigations using a Sievert-type apparatus. The results show that the dehydrogenation pathway of LiBH_4_/*x*LiAlH_4_ had a four-step character. The experimental dehydrogenation amount did not reach the theoretical expectations, because the products such as AlB_2_ and LiAl formed a passivation layer on the surface of Al and the dehydrogenation reactions associated with Al could not be sufficiently carried out. Kinetic investigations discovered a nonlinear relationship between the activation energy (E_a_) of dehydrogenation reactions associated with Al and the ratio *x*, indicating that the E_a_ was determined both by the concentration of Al produced by the decomposition of LiAlH_4_ and the amount of free surface of it. Therefore, the amount of effective contact surface of Al is the rate-determining factor for the overall dehydrogenation of the LiBH_4_/*x*LiAlH_4_ composites.

## 1. Introduction

With the exhaustion of traditional fossil fuels and environmental pollution, the exploration of clean energy is attracting more and more attention. Hydrogen is recognized as one of the most promising clean energy sources. However, the lack of efficient hydrogen storage materials is a formidable problem that hinders the practical application of hydrogen [1,2,3].

There are three main types of hydrogen storage materials that are currently of concern: adsorption hydrogen storage materials (such as activated carbon, mesoporous silica, metal-organic frameworks, etc.) [4,5,6,7], nanostructured hydrogen storage materials (such as carbon nanotubes, fullerene, functionalized adsorption material and nanosized complex hydrides, etc.) [8,9,10,11,12,13,14,15] and coordination hydride hydrogen storage materials (such as LiBH_4_, NaAlH_4_, Mg(NH_2_)_2_, etc.) [16,17,18,19,20]. Among the coordination hydrides, LiBH_4_ is of special interest as materials for solid-state hydrogen storage due to its high theoretical hydrogen storage capacity of 18.5 wt.%. Unfortunately, LiBH_4_ is thermodynamically stable and the conditions for dehydrogenating LiBH_4_ are very harsh. It’s reported that heating LiBH_4_ to 720 °C in an inert atmosphere of argon will result in the following reaction. First, LiBH_4_ will transform from the orthorhombic phase (*o*-LiBH_4_) to the hexagonal phase (*h*-LiBH_4_) at 117 °C. The *h*-LiBH_4_ will then melt at 289 °C. The molten *h*-LiBH_4_ will finally undergo a two-step dehydrogenation reaction based on Equations (1) and (2) at 400–500 °C and 720 °C, respectively [21,22].

LiBH_4_ → LiH + B + 3/2H_2_  (400–500 °C; 13.8 wt.% H_2_) (1)

LiH → Li + 1/2H_2_    (720 °C; 4.7 wt.% H_2_) (2)

In practical applications, only the first step dehydrogenation is usually considered because the temperature required for the second step dehydrogenation is too high. Some researchers have been looking for ways to improve the hydrogen desorption properties of LiBH_4_ and found that it is possible to change the dehydrogenation steps of LiBH_4_ by adding some metals or metal hydrides (such as Mg, Al, Ti, MgH_2_, CaH_2_, etc.) to form metal borides [23,24]. Among these studies, the addition of Al can significantly improve the hydrogen storage performance of LiBH_4_, and reduce the dehydrogenation temperature to around 300 °C [23,25].

Theoretically, LiAlH_4_ is also an important coordination hydride hydrogen storage material. When heated in an inert atmosphere, it will undergo a two-step dehydrogenation reaction based on Equations (3) and (4) respectively [21,26,27], without considering the decomposition of LiH.

3LiAlH_4_ → Li_3_AlH_6_ + 2Al + 3H_2_  (~160 °C; 5.3 wt.% H_2_) (3)

Li_3_AlH_6_ → 3LiH + Al + 3/2H_2_   (210 °C; 2.6 wt.% H_2_) (4)

The practical hydrogen storage capacity of LiAlH_4_ is only 7.9 wt.%, and the reversibility of Li_3_AlH_6_ to LiAlH_4_ is thermodynamically impossible in mild temperature and pressure condition [26]. So LiAlH_4_ is not suitable for hydrogen storage alone. Some researchers [28,29] proposed that it is possible to promote the hydrogen desorption performance of LiBH_4_ by combining with LiAlH_4_. However, there are few reports on the dehydrogenation mechanism and the activation energy of the dehydrogenation steps of LiBH_4_/LiAlH_4_ composites. Therefore, the LiBH_4_/*x*LiAlH_4_ (*x* = 0.5, 1, 2) composites were synthesized by mechanical ball milling using LiBH_4_ and LiAlH_4_ as raw materials. The hydrogen desorption properties of the LiBH_4_/*x*LiAlH_4_ composites were explored systematically, along with kinetic investigations using a Sievert-type apparatus. Kissinger method was used to calculate the activation energy of dehydrogenation steps and the dehydrogenation mechanism was also discussed.

## 2. Materials and Methods

The LiBH_4_ powder (95% purity; Acros Organics, Geel, Belgium) and LiAlH_4_ powder (97% purity; Alfa Aesar, Ward Hill, MA, USA) were used as raw materials in this study. All of the samples were handled in a Mikrouna glove box filled with high purity argon (99.999%) and controlled H_2_O (<0.5 ppm) and O_2_ (<0.1 ppm) concentrations for preventing contamination. LiBH_4_ powders and LiAlH_4_ powders were mixed at a molar ratio of 1:0.5, 1:1, 1:2 respectively, and the LiBH_4_/*x*LiAlH_4_ (*x* = 0.5, 1, 2) composites were synthesized by ball-milling using a QM-3SP4 planetary ball mill (Nanjing Nanda Instrument Plant, Nanjing, China). The ball to powder ratio was 45:1. The milling process was carried out at 400 rpm for 30 min under a 0.1 MPa argon atmosphere. To prevent the temperature from rising too fast during long-term milling, the milling process was paused every 6 min for cooling.

The characterization of hydrogen desorption properties of the LiBH_4_/*x*LiAlH_4_ composites was carried out on a Sieverts-type apparatus with the volumetric method. In this apparatus, a sample chamber is connected to a gas reservoir chamber through a valve, and the internal volumes of both chambers are calibrated before each test. The sample chamber is equipped with a standard K-type thermocouple to directly measure the temperature of samples. The reservoir chamber is also instrumented with a K-type thermocouple to measure the gas temperature, as well as two pressure sensors to monitor the gas pressure. During tests, the temperature and pressure data were automatically collected by a computer, and then the amounts of hydrogen desorbed from samples were calculated by the ideal gas equation (PV = nRT) using the obtained data. The morphologies of the as-prepared LiBH_4_/LiAlH_4_ composite before and after ball-milling were observed by a field emission scanning electronic microscopy (SEM, Hitachi, Tokyo, Japan), and elemental mapping of Al and B in the sample after ball-milling was conducted using an assorted energy-dispersive spectrometer (EDS, Bruker, Karlsruhe, Germany). The thermal events during the dehydrogenation of the samples were investigated by thermogravimetry/differential scanning calorimeter (TG/DSC, Netzsch STA449F3). During measurements, the samples were heated gradually with a set heating rate under flowing argon of 50 mL/min, and the hydrogen desorption spectra were collected synchronously using a mass spectrometer (MS, Netzsch QMS403C). The phase of the as-prepared LiBH_4_/*x*LiAlH_4_ (*x* = 0.5, 1, 2) composites and the dehydrogenation product of them at various temperatures were identified by X-ray diffraction technique (XRD, X’Pert Pro, Cu-Kα, 3 kW). During XRD measurements, the samples were sealed with a polypropylene membrane to avoid exposure to any moisture or oxygen.

## 3. Results and Discussion

### 3.1. Dehydrogenation Performance of LiBH_4_/xLiAlH_4_ Composites

**Figure 1:**
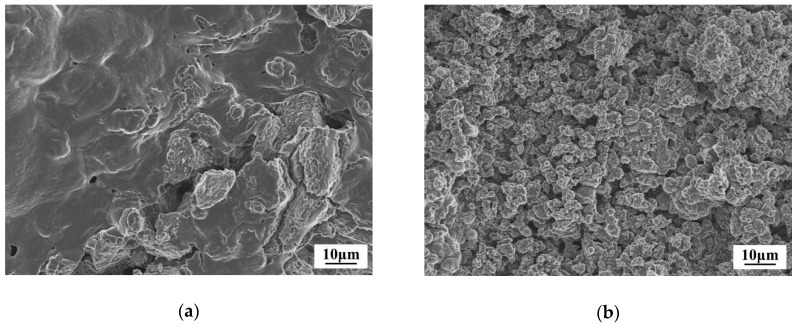

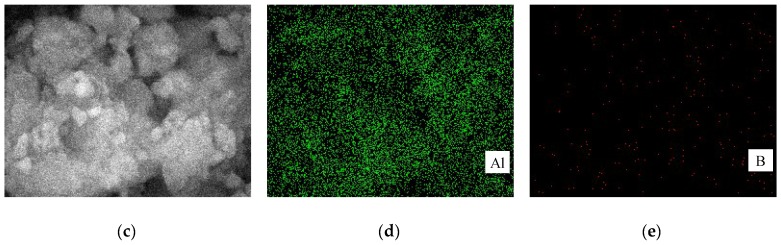
SEM images of LiBH_4_/LiAlH_4_ mixture before (**a**) and after (**b**) ball-milling and micro-selection area (**c**) and elemental mapping of Aluminum (**d**) and Boron (**e**) in the as-prepared LiBH_4_/LiAlH_4_ composite.

**Figure 2:**
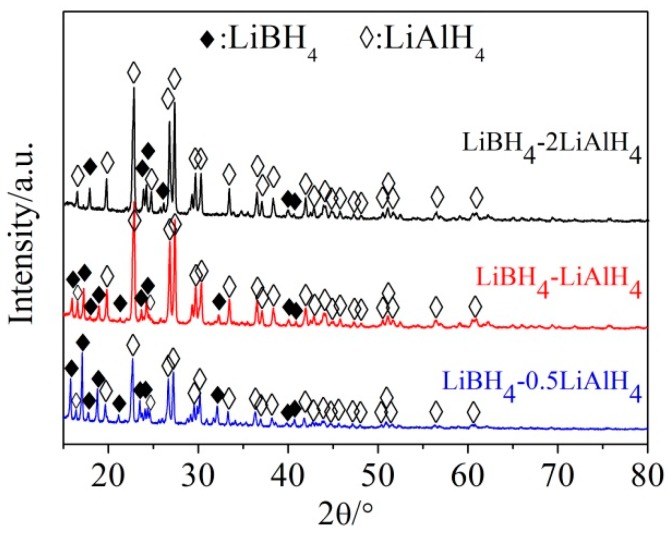
XRD patterns of the as-prepared LiBH_4_/*x*LiAlH_4_ (*x* = 0.5, 1, 2) composites.

The SEM images of the mixture of LiBH_4_ and LiAlH_4_ before and after ball milling at a molar ratio of 1:1 and the elemental mapping of Al and B in the as-prepared LiBH_4_/LiAlH_4_ composite are shown in Figure 1. It can be seen that the particle size of the mixture was dramatically reduced after ball-milling. The Al element representing LiAlH_4_ is uniformly distributed in the LiBH_4_/LiAlH_4_ sample and the signal of B element representing LiBH_4_ is weak but also relatively uniform. The low intensity of the B signal is attributed to the fact that B is a light element, which can only be qualitatively analyzed rather than quantitatively analyzed by the EDS. Based on the above analysis, it can be verified that LiBH_4_ and LiAlH_4_ have been uniformly mixed. Figure 2 demonstrates XRD patterns of the as-prepared LiBH_4_/*x*LiAlH_4_ (*x* = 0.5, 1, 2) composites. As can be seen, only LiBH_4_ and LiAlH_4_ were detected. It indicates that the ball milling process not only achieved the purpose of thoroughly mixing the LiBH_4_ and LiAlH_4_ powders but also prevented them from dehydrating or decomposing before the dehydrogenation test. In addition, the results also show that the diffraction intensity of LiAlH_4_ increases with its content in the samples, which is consistent with the rule that the intensity of diffraction peaks varies with the relative amount of the composition in the composites.

**Figure 3:**
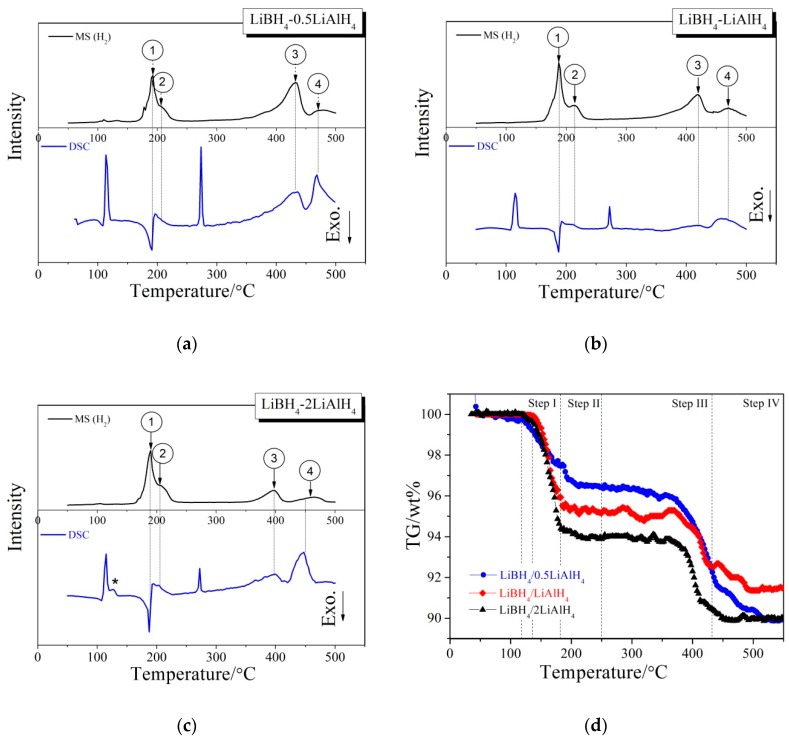
DSC/MS curves of the LiBH_4_/0.5LiAlH_4_ (**a**), LiBH_4_/LiAlH_4_ (**b**), LiBH_4_/2LiAlH_4_ (**c**) and TG curves (**d**) of the LiBH_4_/*x*LiAlH_4_ (*x* = 0.5, 1, 2) samples.

Figure 3 presents different simultaneous signals for the dehydrogenation of LiBH_4_/*x*LiAlH_4_ (*x* = 0.5, 1, 2) composites: the DSC signal, the hydrogen signal and the signal of thermogravimetry (TG) plotted over the temperature. Under an argon atmosphere, the samples were heated from room temperature to 500 °C at a rate of 5 °C/min during measurements. Firstly, the sharp endothermic peak in the DSC curve at 115 °C was attributed to the crystal transformation from orthorhombic phase (*o*-LiBH_4_) to hexagonal phase (*h*-LiBH_4_), while the other sharp endothermic peak at 288 °C was caused by the melting of *h*-LiBH_4_. This is in good agreement with the results reported by other researchers [21,30]. Then four dehydrogenation peaks, denoted 1, 2, 3 and 4 can be observed in the MS curve. The peak 1 and 2 appear in the range of 150 °C to 250 °C and partially overlap, while the peak 3 and 4 appear in the range of 350 °C to 500 °C. The peak 1, 2, 3 and 4 correspond to one exothermic peak and three endothermic peaks in the DSC curve respectively, indicating that the dehydrogenation has a four-step character. In addition, the endothermic peak labeled “*” in Figure 3c corresponds to the melting of LiAlH_4_ [31]. This peak is not obvious in the DSC curves of LiBH_4_/0.5LiAlH_4_ and LiBH_4_/LiAlH_4_ composites (Figure 3a,b), because the relative amounts of LiAlH_4_ in these two samples are low, and the melting endothermic peak of LiAlH_4_ overlaps with the crystal transformation endothermic peak of LiBH_4_. Figure 3d displays the TG curves of LiBH_4_/*x*LiAlH_4_ (*x* = 0.5, 1, 2) samples. It can be seen that the dehydrogenation process of each sample can be divided into four steps, corresponding to the four dehydrogenation peaks denoted 1, 2, 3 and 4 in Figure 3a–c. The initial dehydrogenation temperature of LiBH_4_/0.5LiAlH_4_ and LiBH_4_/2LiAlH_4_ is about 117 °C and that of LiBH_4_/LiAlH_4_ is approximate 138 °C. All of them are lower than the dehydrogenation temperature (~160 °C) of pure LiAlH_4_. It may be due to the fact that the LiBH_4_/*x*LiAlH_4_ particles become much smaller after ball milling, which reduces the activation energy of the first-step reaction of dehydrogenation. The total dehydrogenation amount of each LiBH_4_/*x*LiAlH_4_ (*x* = 0.5, 1, 2) sample at 500 °C reached 9.6 wt.%, 8.7 wt.% and 10.2 wt.%, respectively. Other possible gas products or impurities (such as B_2_H_6_, etc.) were also examined by the MS and no other gas species was detected. It suggests that the weight loss of all three samples is caused by releasing pure H_2_.

### 3.2. Mechanism of the Dehydrogenation Process

**Figure 4:**
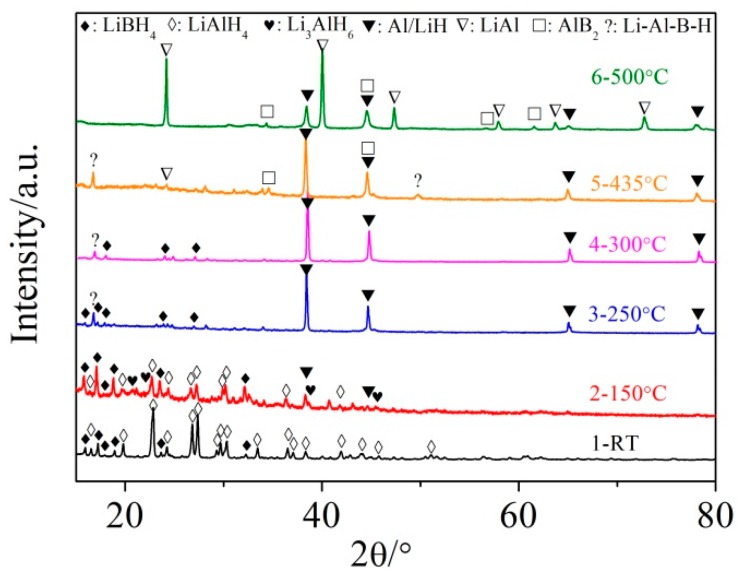
XRD patterns of the LiBH_4_/LiAlH_4_ composite obtained at different temperatures (room temperature, 150, 250, 300, 435 and 500 °C).

In order to investigate the hydrogen desorption mechanism of the LiBH_4_/*x*LiAlH_4_ (*x* = 0.5, 1, 2) composites, XRD analysis was conducted on the solid products of LiBH_4_/LiAlH_4_ sample at different dehydrogenation temperatures (e.g. 150 °C, 250 °C, 300 °C, 435 °C and 500 °C), which was indicated by the DSC curve (Figure 3b). The results are shown in Figure 4. It can be seen that the diffraction peaks of LiBH_4_ barely changed and no boron (B)-related phases were detected when the sample was heated to 150 °C. However, the diffraction intensity of LiAlH_4_ visibly decreased and a few diffraction peaks of Li_3_AlH_6_ and Al appeared (the diffraction peaks of Al and LiH overlapped). It indicates that LiAlH_4_ decomposed to form Li_3_AlH_6_ and Al at 150 °C and LiBH_4_ was not involved in the reaction. Combining with the results of Figure 3b, this reaction was exothermic and gave off H_2_, which is in good agreement with the decomposition reaction of LiAlH_4_ (Equation (3)).

The diffraction peaks of LiAlH_4_ and Li_3_AlH_6_ disappeared and the diffraction intensity of LiBH_4_ decreased when the sample was heated to 250 °C. At the same time, the diffraction intensity of Al and LiH increased significantly, which means that LiAlH_4_ and Li_3_AlH_6_ had been completely decomposed to LiH, Al and H_2_ (the peak 2 in the MS curve of Figure 3b). Thus the second step dehydrogenation of LiBH_4_/LiAlH_4_ composite was assumed to be associated with the decomposition of Li_3_AlH_6_ based on Equation (4).

The theoretical dehydrogenation amounts of Equations (3) and (4) were 3.4 wt.% and 1.7 wt.%. However, the experimental dehydrogenation amounts of the first and second step were 4.0 wt.% and 1.0 wt.%, respectively. The actual dehydrogenation amount of the first step is more than the theoretical amount of Equation (3), while that of the second step is less than the theoretical amount of Equation (4). Even so, the experimental value of the total dehydrogenation capacity (5.0 wt.%) in the range of 150 °C to 250 °C is very close to the theoretical value (5.1 wt.%) based on Equations (3) and (4). It can be attributed to the partial overlap between these two reactions. That is to say, some of Li_3_AlH_6_ had begun to decompose when LiAlH_4_ was not completely converted to Li_3_AlH_6_ in the first dehydrogenation step, which led to an increase of the hydrogen released. The remaining Li_3_AlH_6_ was decomposed in the second step of dehydrogenation, resulting in a lower hydrogen release than the theoretical value. Therefore, the first and second step dehydrogenation can be ascribed to reactions based on Equations (5) and (6), respectively.

LiAlH_4_ → 1/3Li_3_AlH_6_ + 2/3Al + H_2_, α(1/3Li_3_AlH_6_) → α(LiH + 1/3Al+ 1/2 H_2_), 0 < α < 1 (5)

(1 − α)(1/3Li_3_AlH_6_) → (1 − α)(LiH + 1/3Al + 1/2H_2_), 0 < α < 1 (6)

It also should be mentioned that a tiny diffraction peak that cannot be identified, marked “?”, appeared at 250 °C. Similar unknown phases were also reported by other researchers and suggested to be an intermediate product from the reaction of LiBH_4_ and Al [32,33,34]. This may be also the reason for the decrease of the diffraction intensity of LiBH_4_. After the LiBH_4_/LiAlH_4_ sample was heated from 250 °C to 300 °C there was no obvious change in phase composition. A slight fluctuation in the TG curve of LiBH_4_/LiAlH_4_ was observed within this temperature range, which caused by the melting of LiBH_4_.

The diffraction peaks of LiBH_4_ did not disappear until the sample was heated to 435 °C, followed by the appearance of AlB_2_ and a tiny diffraction peak of LiAl. The peaks of Al, LiH and the intermediate compound remained. According to the work of other researchers [32], LiBH_4_ reacted with Al to form LiH and AlB_2_ and gave off H_2_ at this stage based on Equation (7), which is the main reason for the dive of the TG curve between 350 °C and 435 °C. And LiH began to react with Al to produce LiAl and H_2_ based on Equation (8), which is the reason why the TG curve of LiBH_4_/LiAlH_4_ has an inflection point around 435 °C. When the sample was heated to 500 °C, the diffraction peaks of LiH and Al were obviously weakened and the intermediate compound disappeared. However, the diffraction intensity of LiAl increased noticeably. This phenomenon can be attributed to the decomposition of an intermediate compound and the proceedings of reaction based on Equation (8). The temperature ranges of the above reactions are in good agreement with those reported by other researchers [23].

LiBH_4_ + 1/2Al → LiH + 1/2AlB_2_ + 3/2H_2_ (7)

LiH + Al → LiAl + 1/2H_2_ (8)

Similarly, the theoretical dehydrogenation amounts of Equations (7) and (8) were 5.0 wt.% and 0.9 wt.%, while the experimental dehydrogenation amounts of the third and fourth step were 2.4 wt.% and 1.3 wt.%, respectively. On the hand, the reaction between LiBH_4_ and Al formed an intermediate compound (denoted “Li-Al-B-H”) first and then decomposed to liberate H_2_ (Equation (9)). Incomplete decomposition of intermediate compounds leads to insufficient dehydrogenation in the third step. The remaining intermediate compound was decomposed in the fourth step, resulting in an increase in the amount of dehydrogenation. On the other hand, the total amount of dehydrogenation in the third and fourth steps is much less than that of theoretical dehydrogenation based on Equations (7) and (8). This may indicate that some kinetic barriers exist in the above dehydrogenation reactions. The physical barrier is likely to be the reaction products such as “Li-Al-B-H” compound, AlB_2_ and LiAl, which act as passivating layers that preventing Al from contacting with LiBH_4_ and LiH. The dehydrogenation reactions associated with Al were not performed sufficiently, resulting in the total amount of hydrogen released not as high as the theoretical value. This can also be supported by the fact that amounts of LiH and Al can still be detected by XRD even at 500 °C.

2LiBH_4_ + Al → “Li-Al-B-H” → *β*(AlB_2_ + 2LiH + 3H_2_) + (1 − *β*) “Li-Al-B-H”, 0 < *β* < 1 (9)

The whole hydrogen desorption process of the LiBH_4_/LiAlH_4_ sample can be concluded as follows: As heating in the crucible, LiBH_4_ first transformed from orthorhombic phase (*o*-LiBH_4_) to hexagonal phase (*h*-LiBH_4_) at around 112 °C. Then LiAlH_4_ started to decompose into Li_3_AlH_6_ and Al and liberated H_2_ based on Equation (3) near 150 °C. Before LiAlH_4_ was completely converted, the product Li_3_AlH_6_ began to decompose into LiH and Al while releasing H_2_ based on Equation (4). These two reactions partially overlapped in the range of 150 °C to 250 °C. And an intermediate compound from the reaction of LiBH_4_ and Al appeared near 250 °C. As the temperature rose, LiBH_4_ melted and then reacted with Al, through intermediate compound containing “Li-Al-B-H”, to form LiH and AlB_2_ and gave off H_2_ based on Equation (9). The “Li-Al-B-H” compound did not completely decompose in the range of 350 °C to 435 °C and the product LiH began to react with Al to produce LiAl and H_2_ based on Equation (8) near 435 °C. When the sample was heated to 500 °C, the remaining “Li-Al-B-H” compound was completely decomposed and the reaction between LiH and Al proceeded to the maximum extent. The experimental dehydrogenation amount of LiBH_4_/LiAlH_4_ sample was not as high as the theoretical expectations, because the reaction products such as AlB_2_ and LiAl formed a passivation layer on the surface of Al and the dehydrogenation reactions associated with Al could not be sufficiently carried out. Therefore, the amount of free Al surface is the rate-determining factor for the overall dehydrogenation of the LiBH_4_/LiAlH_4_ composites.

### 3.3. Kinetic Investigations of the Dehydrogenation Reaction

**Figure 5:**
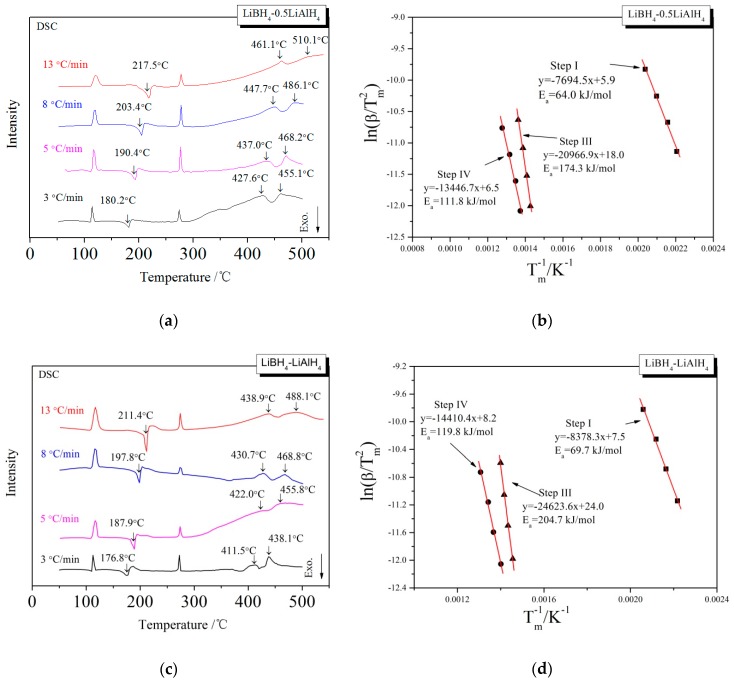

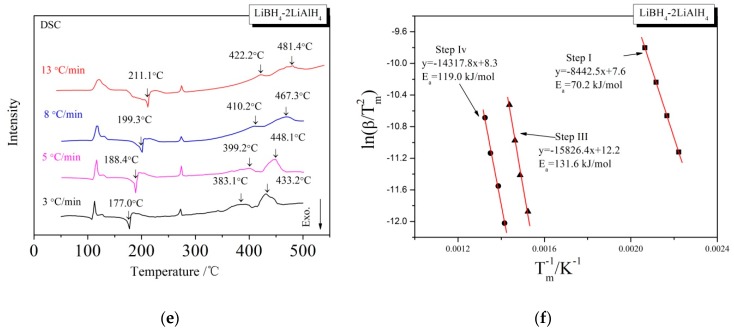
DSC curves (**a**,**c**,**e**) at different heating rates and the Kissinger spectra (**b**,**d**,**f**) that ln(β/T_m_^2^) as a function of T_m_^−1^ for the decomposition steps of the LiBH_4_/*x*LiAlH_4_ (*x* = 0.5, 1, 2) samples.

In order to investigate the kinetic properties of the dehydrogenation of LiBH_4_/*x*LiAlH_4_ (*x* = 0.5, 1, 2) composites, Kissinger method [35] was used to calculate the activation energy (E_a_) of dehydrogenation reaction. The E_a_ of the dehydrogenation reaction at each step can be obtained from the slope of a linearly fitted line in the ln(β/T_m_^2^) − T_m_^−1^ spectrum, where β is the heating rate and T_m_ is the dehydrogenation temperature shown in the DSC curve. The LiBH_4_/*x*LiAlH_4_ samples were heated to 500 °C at the rates of 3, 5, 8 and 13 °C/min, respectively. The DSC curves at various heating rates and the Kissinger spectra reflecting the E_a_ of the first, third and fourth step of dehydrogenation are shown in Figure 5. The calculation of the E_a_ of the second step of dehydrogenation is not included because the corresponding endothermic peak is not obvious in the DSC curves, and it is difficult to determine the accurate dehydrogenation temperature T_m_. The E_a_ of the first, third and fourth steps of the dehydrogenation of LiBH_4_/*x*LiAlH_4_ (*x* = 0.5, 1, 2) samples are listed in Table 1. It can be seen that the E_a_ of the first step is in the range of 64.0–70.2 kJ/mol, which is lower than the 82 kJ/mol reported by Andreasen et al. [36]. It is probably due to the decrease of size and increase of surface energy of the LiAlH_4_ particles after ball milling, which leads to the decrease of E_a_ of the LiAlH_4_ decomposition reaction. The E_a_ of the dehydrogenation reaction in the third step is in the range of 131.6–204.7 kJ/mol, while the E_a_ in the fourth step is in the range of 111.8–119.8 kJ/mol. They both increase first and then decrease as the ratio coefficient *x* increases from 0.5 to 2, which is in good agreement with the variation of dehydrogenation capacity with the ratio *x*. The dehydrogenation reactions in the third and fourth step both associated with Al. The nonlinear relationship between the apparent activation energy and the ratio *x* is due to that the E_a_ is affected both by the concentration of Al produced by the decomposition of LiAlH_4_ and the amount of free surface of it. When the ratio *x* increased from 0.5 to 1, the effective contact area between Al and other reactants (LiBH_4_, LiH, etc.) decreased instead may be due to severer agglomeration or passivation layer of it. So the activation energy required for the reaction increased. When the ratio *x* further increased from 1 to 2, the beneficial effect of increasing the concentration of Al exceeded the adverse effect of aggregation or passivation layer. The effective contact area increased and the activation energy required for the reaction decreased.

## 4. Conclusions

The dehydrogenation pathway of LiBH_4_/*x*LiAlH_4_ (*x* = 0.5, 1, 2) composites had a four-step character: The first step involved the decomposition of LiAlH_4_ to form Li_3_AlH_6_, Al and H_2_ and partial decomposition of Li_3_AlH_6_ to form LiH and Al while releasing H_2_. The remaining Li_3_AlH_6_ was decomposed in the second step. Then LiBH_4_ reacted with Al, through an intermediate compound containing “Li-Al-B-H”, to form LiH and AlB_2_ and gave off H_2_. The “Li-Al-B-H” compound did not completely decompose in the third step and the product LiH began to react with Al to produce LiAl and H_2_. Finally, the remaining “Li-Al-B-H” compound was completely decomposed and the reaction between LiH and Al proceeded to the maximum extent in the fourth step. The experimental dehydrogenation amount of LiBH_4_/LiAlH_4_ did not reach the theoretical expectations, because the products such as AlB_2_ and LiAl formed a passivation layer on the surface of Al and the dehydrogenation reactions associated with Al could not be sufficiently carried out. 

The kinetic investigations of the dehydrogenation showed a nonlinear relationship between the activation energy (E_a_) of the third and fourth step and the ratio *x*, indicating that the E_a_ was determined both by the concentration of Al produced by the decomposition of LiAlH_4_ and the amount of free surface of it. Therefore, the amount of free Al surface is the rate-determining factor for the overall dehydrogenation of the LiBH_4_/*x*LiAlH_4_ composites. 

Further work has to focus on identifying the unknown “Li-Al-B-H” compound appearing in the dehydrogenation reactions as well as on cracking the kinetic barriers to improve the hydrogen desorption properties of LiBH_4_/*x*LiAlH_4_ system.

## Figures and Tables

**Table 1:** The activation energy (E_a_) of the first, third and fourth dehydrogenation steps of LiBH_4_/*x*LiAlH_4_ (*x* = 0.5, 1, 2) samples.

*x*	E_a_ of the First Step (kJ/mol)	E_a_ of the Third Step (kJ/mol)	E_a_ of the Fourth Step (kJ/mol)
0.5	64.0	174.3	111.8
1	69.7	204.7	119.8
2	70.2	131.6	119.0
